# Serum Soluble Vascular Cell Adhesion Molecule-1 Overexpression Is a Disease Marker in Patients with First-Time Diagnosed Antinuclear Antibodies: A Prospective, Observational Pilot Study

**DOI:** 10.1155/2018/8286067

**Published:** 2018-02-01

**Authors:** Mara Oleszowsky, Matthias F. Seidel

**Affiliations:** ^1^Praxis für Rheumatologie, Köln, Germany; ^2^Medizinische Klinik III, Hematology, Oncology and Rheumatology, University Hospital of Bonn, Bonn, Germany; ^3^Schmerzklinik Basel, Basel, Switzerland

## Abstract

**Objective:**

Antinuclear antibodies (ANA) serve as screening tests for connective tissue diseases but have low specificity. In this pilot study, we aimed to identify patients with first-time positive ANA and musculoskeletal complaints and correlate serum soluble vascular adhesion molecules as biomarkers.

**Methods:**

Prospective, observational study with 100 ANA-positive patients, comparing them to age- and gender-matched healthy controls (HC, *n* = 75), was conducted. Serum levels of soluble intercellular adhesion molecule-1 (sICAM-1), endothelial-leukocyte adhesion molecule-1 (sELAM-1), and vascular cell adhesion molecule-1 (sVCAM-1) were measured. A subgroup of patients with systemic sclerosis (SSc) treated with immunosuppressants was followed over 10 months.

**Results:**

Patients belonged to three main entities: rheumatoid arthritis (RA, *n* = 32), collagen diseases (CD, *n* = 56) also including systemic sclerosis (SSc, *n* = 11), and other autoimmune diseases (*n* = 12). sICAM-1 was similar among groups. sELAM-1 was elevated by 1.9-fold in only in SSc. sVCAM-1 was elevated by 3.1-fold in RA and by 3.3-fold in CD and in other autoimmune diseases by 3.4-fold. Seven SSc patients with immunosuppression had a 2.7-fold increased sVCAM-1 at baseline and reached the levels of healthy controls after 5 months, while CRP, ESR, and clinical parameters remained unchanged.

**Conclusion:**

Our study suggests that sVCAM-1 is a disease marker independent of standard serum parameters in several rheumatic diseases. This study is registered with EU PAS Register number: EUPAS22154.

## 1. Introduction

Serum antinuclear antibodies (ANA) are the classical screening parameter for collagen diseases (CD), but they are also found in patients with rheumatoid arthritis (RA), other autoimmune diseases, and virus infections and also in healthy individuals [1, 2]. Thus, ANA have low specificity and usually a titer of 1 : 160 is considered as positive [3]. Most importantly, the presence of ANA in serum is significant only in combination with clinical symptoms. ANA are most frequently found in CD, such in systemic lupus erythematosus (SLE), systemic sclerosis (SSc), Sjögren's syndrome (SjS), RA, and others [4, 5]. The different forms of CD may share a common vasculitis background. For example, a clear relationship is found between the progression of nailfold capillaries with endothelial pathology and ANA patterns in SSc [6]. In addition, vasculopathy and disordered angiogenesis are found in RA and SSc [7] with a clear predominance for the latter. Thus, molecules linked to endothelial pathology might be useful indicators of disease activity and perhaps for selecting an appropriate therapeutic intervention.

Several soluble isoforms of endothelial adhesion molecules have been studied in rheumatic diseases. Important markers include the soluble isoforms of intercellular adhesion molecule-1 (sICAM-1), endothelial-leukocyte adhesion molecule-1 (sELAM-1), and vascular cell adhesion molecule-1 (sVCAM-1). These molecules mediate transendothelial migration, and, thus, they are upregulated during autoimmune activation [8]. VCAM-1 and ICAM-1 induce adhesion of lymphocytes, monocytes, eosinophils, and basophils to vascular endothelium. VCAM-1 is expressed by activated endothelial cells, renal tubular epithelial cells, dendritic cells, and macrophages [9–11]. ELAM-1, on the other hand, is only found on activated endothelium and fibroblasts [12].

VCAM-1 and ICAM-1 are receptor-like, membrane-bound proteins and belong to the immunoglobulin-like superfamily. In contrast, ELAM-1 belongs to the selectins, a distinct group of adhesion molecules. During inflammation, ELAM-1 plays an important role in recruiting leukocytes to the site of injury [13]. Upregulation of adhesion molecules in endothelial cells is stimulated by cytokines, like tumor necrosis factor- (TNF-) *α* or interleukin-1 [14]. Soluble adhesion molecules in serum therefore may be useful indicators for endothelial activation and inflammation, for example, in evaluating SSc [15].

Increased serum levels of adhesion molecules have been described in many different rheumatic diseases. For example, sICAM-1 was elevated in patients with giant cell arthritis, and it was correlated with disease activity [16]. Increased concentrations of ELAM-1, ICAM-1, and VCAM-1 were found in affected skin from patients with SSc. Moreover, the highest levels were present in the diffuse form of SSc, indicating that these proteins may be involved in the early stages of tissue fibrosis [17]. Upregulated sVCAM-1 was found in SLE, SSc, and RA [18–20]. Similarly, elevated sVCAM-1, sICAM-1, and sELAM-1-1 were also detected in patients with RA, SSc, and vasculitis [21]. Another study showed elevated sICAM-1 in patients with SSc [22]. sICAM-1, sVCAM-1, and sELAM-1 activities were correlated with clinical disease activity in patients with SSc [23].

Vascular dysfunction is considered to be one of the earliest and most crucial initiating events in the pathogenesis of CD such as SSc [24] suggesting that serum soluble vascular adhesion markers may be of diagnostic significance. In SSc, routine measurements of erythrocyte sedimentation rates (ESRs) or C-reactive protein (CRP) levels are frequently normal [25] and thus markers for CD activity are warranted.

This prospective observational pilot study aimed to identify serum markers for ANA-positive diseases. At first, we selected groups of patients with musculoskeletal complaints and first-time positive ANA detection. We then measured sets of individual serum markers (i.e., sELAM-1, sVCAM-1, and sICAM-1), related them to clinical and standard serological measures, and compared them to age- and gender-matched healthy controls (HC). Third, focusing on SSc, we determined whether putatively elevated serum markers decrease during immunosuppressive pharmacotherapy.

## 2. Methods

### 2.1. Patients and Healthy Control

This study was approved by the local ethics committee. All patients who provided written, informed consent were included. A total of 127 patients with musculoskeletal complaints were screened. For study eligibility, positive ANA had to be present, defined as a titer of at least 1 : 160. A total of 100 patients were finally included; 27 patients did not sign the informed consent. Patients underwent a routine physical examination, in-house laboratory blood analyses of ESR, CRP, blood count, routine liver and kidney parameters, and urine analysis. In case of patients with SSc, study parameters were also analyzed over a 10-month period.

### 2.2. Other Laboratory Assays

Serum ANA were assessed by indirect immunofluorescence in HEp-2 cells (Euroimmun, Germany). Rheumatoid factor (RF) was measured in immunoassays with the Dimension Vista® System (Siemens Healthcare Diagnostics, Germany). Anti-neutrophil cytoplasmatic antibodies (ANCA) were assessed with indirect immunofluorescence in a composite substrate of ethanol and formalin-fixed granulocytes (EOH) and HEp-2 cells, (Euroimmun, Germany). Anti-double-stranded DNA (dsDNA) antibodies were measured with an anti-dsDNA-NcX-ELISA (Euroimmun, Germany).

Serum concentrations of human sICAM-1 (DY720), sELAM-1 (DY72), and sVCAM-1 (DY809) were analyzed by commercially available ELISA (Duo-Set-Kit (R&D Systems, Germany). Reagent diluent concentrate 2 (DY995) and the substrate reagent Pack (DY999) were also purchased from R&D Systems. Sulphuric acid solution (35276) and phosphate buffered saline (P4417) were purchased from Sigma Aldrich (Germany). High binding 96-well ELISA plates (655081) were obtained from Greiner (Germany). Soluble adhesion molecule parameters for age- and gender-matched HC (*n* = 75) were determined in sera from healthy blood donors, after obtaining written, informed consent.

### 2.3. Additional Diagnostics

We also recorded data on premedications, comorbidities, Health Assessment Questionnaire (HAQ) scores, visual analogue scale (VAS; range: 0–100) for pain, patient and physician global assessment (PtGA and PGA, resp.), and modified-Rodnan-Skin scores [26]. Diagnoses were made according to international classification criteria, where possible, and subdivided into three main groups. The first group consisted of patients with RA. The second group comprised CD. Finally, the third group included other autoimmune diseases. The classification criteria included the American College of Rheumatology/European League Against Rheumatism (ACR/EULAR) criteria for RA (2010) [27], ACR/EULAR criteria for the classification of SSc [28]; ACR criteria for SLE [29]; criteria for classifying SjS from the revised European criteria [30]; and undifferentiated connective tissue diseases (CTD) [31]. Clinical diagnostics also included documentation of the presence or absence of Raynaud's phenomenon (RP), puffy fingers (PFs), and digital ulcers (DUs). Additional diagnostic measures comprised the determination of pulmonary arterial pressure (PAP) determined by echocardiography (ECHO); lung function: diffusion capacity of the lung for carbon monoxide/alveolar volume, transfer coefficient, Krogh index (DLCO/VA), forced expired volume in one second (FEV1), and vital capacity (VC). None of the patients had estimated mean PAP findings above 40 mmHg.

Seven patients with SSc required immunomodulatory therapy with prednisone (*n* = 4), antimalarials (*n* = 2), methotrexate (*n* = 1), leflunomide (*n* = 2), or mycophenolate-mofetil (*n* = 1). Only these patients were analyzed at baseline and after 3, 5, and 10 months of therapy. Measurements also included the VAS, HAQ, PtGA, ESR, C3/C4, ANA, ANCA, anti-dsDNA antibodies, CRP, RF, anti-citrullinated protein antibodies, sVCAM-1, sICAM-1, and sELAM-1. After 5 and 10 months of therapy, we determined additional ECHO parameters, lung function, pulmonary diffusion capacity, and Rodnan skin score.

### 2.4. Statistics

100 patients and 75 age- and gender-matched HCs were analyzed. 25 HCs were matched at a 1 : 2 ratio with patients of the same gender and age. The three patient groups (RA, CD, and others) were analyzed as a combined population each. In addition, descriptive statistics was performed for the SSc subgroup analyses, however, due to the small sample size with only limited validity. All data were calculated with GraphPad Prism 7. The independent *t*-test was calculated with GraphPad Prism 6. A *p* value ≤ 0.05 was considered as statistically significant. Data were expressed as the mean with standard deviation (SD) if the normality test was positive and Gaussian distribution was always analyzed. Statistics were only calculated for groups with seven or more patients. ANA titers were expressed as median value.

## 3. Results

### 3.1. Patients and Disease Class Groups

The mean age of patients was 53 ± 14 years, the female : male ratio was 79 : 21. The mean age of HC was 50 ± 12 years. Thirty patients had received steroid and/or immunosuppressant treatments before the first test for the presence of ANA, and 68 patients received immunosuppressive regimens after inclusion into the study. Twenty patients were smokers.

The first patient group (*n* = 32) included RA. The second group with CD (total *n* = 56) involved UCTD (*n* = 30), SSc (*n* = 11), SjS, (*n* = 7), Sharp syndrome (*n* = 5), SLE (*n* = 2), and polymyositis/dermatomyositis (*n* = 1). Patients with SSc could be further subdivided into limited cutaneous SSc (*n* = 4), diffuse cutaneous SSc (*n* = 3), and antimyositis/scleroderma overlap (*n* = 4). Finally, the third group included other autoimmune diseases (total *n* = 12) such as psoriatic arthritis (*n* = 3), multiple sclerosis (*n* = 2), autoimmune hepatitis (*n* = 1), autoimmune thyroiditis (*n* = 1), eosinophilic fasciitis (*n* = 1), primary biliary cirrhosis (*n* = 1), polymyalgia (*n* = 1), pANCA-associated vasculitis (*n* = 1), and virus hepatitis C (*n* = 1).

### 3.2. Clinical Findings

Clinical findings as descriptive subgroup analyses are shown in Table 1. Results for ESR, CRP, ANA, Scl-70/ACA, ECHO, DLCO/VA, RP, HAQ, and VAS were numerically similar for all groups. The SSc group had the highest ANA titers (median 1 : 10240) and the highest percentages of patients with RP (82%) and PAP 30–40 mmHg (27%) as determined by ECHO. None of the patients had a PAP in excess to 40 mmHg, and in the absence of exertional dyspnea right heard catheter was not performed. The Rodnan skin score was 10.5 ± 7.3 at baseline. The RA patient group had the highest HAQ and VAS scores. In the CD group, 30% of patients had a TLCO/VA < 70%. 28% of patients had RP in the RA group.

### 3.3. Analysis of sICAM-1 and sELAM-1

Serum levels of sICAM-1 were similar in patient groups and HC (not shown). In contrast, sELAM-1 (ng/ml) was significantly different in the SSc group (*n* = 11) compared to the HC (5.3 ± 1.9 versus 2.8 ± 2.3, *p* = 0.02).

### 3.4. Analysis of sVCAM-1

**Figure 1:**
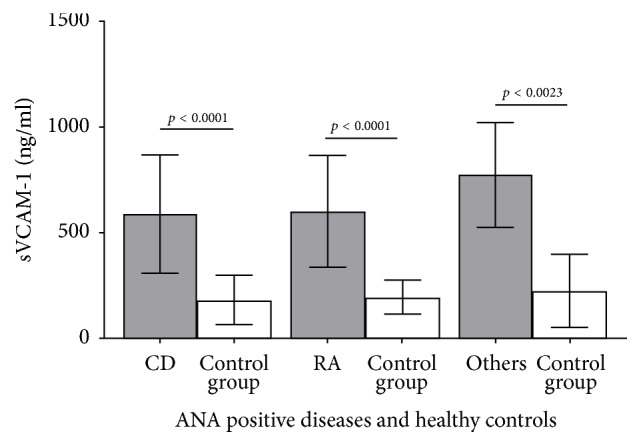
Serum concentrations (ng/ml) of soluble vascular cell adhesion molecule-1 (sVCAM-1). Three patient groups (grey bars) are compared to age- and gender-matched healthy controls (white bars). Whiskers indicate the ± standard deviations. Patient groups consisted of the arthritis group (*n* = 32) with rheumatoid arthritis (RA), the collagen diseases (CD) group involving undifferentiated connective tissue diseases (*n* = 30), systemic sclerosis (*n* = 11), Sjögren's syndrome (*n* = 7), Sharp syndrome (*n* = 5), systemic lupus erythematosus (*n* = 2), and polymyositis/dermatomyositis (*n* = 1). The third group (*n* = 12) included other autoimmune diseases, for example, psoriatic arthritis, multiple sclerosis, and further diseases.

The findings of HC in each group were unequal probably because of the different age- and gender characteristics in each patient group. Serum sVCAM-1 levels were significantly elevated in RA (601.6 ± 264.5 versus 194.2 ± 79.9, *p* < 0.0001), CD (587.7 ± 281.0 versus 179.3 ± 116.8, *p* < 0.0001), and others (775.4 ± 248.2 versus 226.3 ± 174.2, *p* = 0.002). The results are shown in Figure 1.

### 3.5. Subgroup of Patients with SSc with Immunosuppressive Treatment

Seven of the 11 SSc patients were treated with immunosuppressants due to ongoing clinical activity. Two patients were diagnosed with diffuse cutaneous SSc. Of these two patients, one was treated with 250 mg daily chloroquine, due to intense arthralgia; the other received 2 g mycophenolate-mofetil daily due to pulmonary fibrosis (determined with high resolution computed tomography) in combination with a reduction in capillary oxygen saturation (97.4 mmHg at rest and 82.7 mmHg after 50 Watt exercise). The latter patient showed improvements in dyspnea and oxygen saturation at rest and after exercise with treatment. A third patient had systemic limited SSc and received 20 mg leflunomide and 5 mg prednisone daily, due to active arthritis (indicated by bone scan) and bone marrow edema in the right ankle joint (detected with magnetic resonance imaging; not shown). Four of the 7 patients were diagnosed with overlapping symptoms and they were treated for active arthritis with 5 mg prednisone daily, 20 mg methotrexate weekly, 20 mg leflunomide daily, and 200 mg hydroxychloroquine daily, respectively.

**Figure 2:**
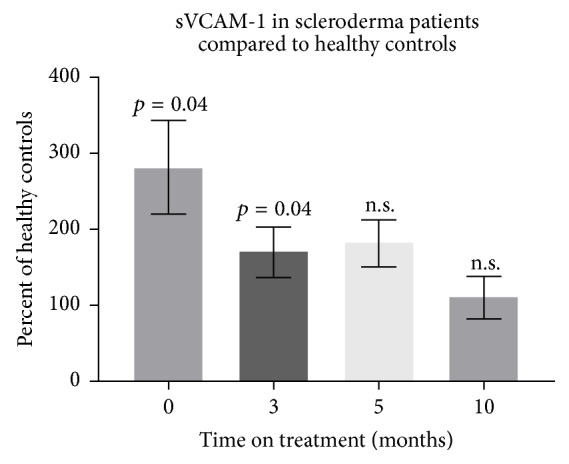
Serum soluble vascular cell adhesion molecule-1 (sVCAM-1) in patients with SSc receiving immunosuppression determined over time shown as the percentage of healthy controls. Baseline, 281.3% ± 61.6; 3 months, 169.8% ± 32.8; 5 months, 181.7% ± 31.6; and 10 months, 110.1% ± 28.3.

In this subgroup of immunosuppressed patients with SSc, sVCAM-1 was also significantly increased at baseline compared to HC (503.7 ± 336.8 ng/l versus 187.1 ± 94.2 ng/l, *p* = 0.04). After therapy, sVCAM-1 levels did not reach the level of HC after 3 months (296.4 ± 139.1 ng/l; *p* = 0.04, paired *t*-test). In contrast, after 5 months (301.1 ± 112.1 ng/l; *p* = 0.06, paired *t*-test) and after 10 months (200.7 ± 102.7 ng/l; *p* = 0.82, paired *t*-test), sVCAM-1 serum concentrations were no more significantly different from HC. The percentages of HC over time are shown in Figure 2. In contrast, immunosuppression did not significantly change the HAQ scores, modified Rodnan total skin scores, VAS scores, ESR, CRP, or other parameters, including TLCO/VA and ECHO (Table 2).

In the same small subgroup, sICAM-1 and sELAM-1 were not significantly elevated before and after therapy.

## 4. Discussion

Soluble serum adhesion molecules such as sVCAM-1 appear as a promising tool for the diagnosis and perhaps monitoring instrument for CD. Up to date, these diseases are identified by ANA [32] that bind to distinct structures within the cell nucleus [33].

CD are heterogeneous diseases with a wide range of visceral complications and diverse skin involvements. Markers are in great demand to identify CD, particularly those with high risk of complications. For example, elevated sVCAM-1 was found in the early and late stages of CD [34–36]. The soluble isoforms of adhesion molecules are thus excellent candidates to indicate disease activity and perhaps therapeutic responses.

Numerous studies have previously described potentially important biomarkers that may provide information about the functional status of endothelial cells and their dysfunction in SSc [37–42]. However, findings of soluble adhesion molecules have been somewhat contradictory up to date. Thus, we aimed to resolve this issue with this prospective observational study by comparing sera of a defined cohort of patients with rheumatic diseases (ANA^+^) and healthy individuals matched for age and gender. Additional subgroup analysis with descriptive statistics also focused on SSc.

We found that sICAM-1 was not significantly different in any of the disease groups. ICAM-1 is expressed by a variety of different cell types which may in part explain the lack of specificity, particularly in patients with SSc [43]. Thus, we conclude that sICAM-1 may not be a relevant disease marker although it was significantly elevated by 1.6-fold in a larger cohort of 33 SSc patients [44]. Our SSc patient group did not show such an increase, perhaps due to a smaller sample volume. Also, comparable to our results, sICAM was not significantly elevated in patients with RA (*n* = 46) and SLE (*n* = 53) [45].

Unlike sICAM-1, sELAM-1 showed an increase in the SSc group. In the subgroup of patients with SSc that received immunosuppressants, sELAM-1 concentrations, however, were not different, probably because of the very small sample size. Based on these results, we conclude that sELAM-1 may have some value as a disease marker, perhaps for patients in the more active stages of SSc. However, these data do not permit a conclusive interpretation and certainly would require a study of a more extensive patient population.

In contrast to sICAM-1 and sELAM-1, we detected a strong signal for sVCAM-1 in all patient groups. These findings suggest that sVCAM-1 might be a robust marker in several groups of rheumatic diseases while CRP and ESR were close to normal. Interestingly, clinical parameters (i.e., VAS, PtGA, and PGA) also showed a 3- to 5-fold elevation in a similar order of magnitude as sVCAM-1 serum concentrations. However, this pilot study was only intended to explore soluble adhesion molecules as biomarkers. A more extensive study would be necessary to clarify if and how sVCAM-1 serum concentrations correlate with additional clinical parameters in selected rheumatic diseases. sVCAM-1 as a biomarker might be a useful tool to objectively identifying the presence of rheumatic diseases. This is most important because sometimes it is difficult if not impossible to define a somatic cause of symptoms in the absence of serological markers.

sVCAM-1 serum concentrations were also observed in the SSc group and a robust decline was observed with immunosuppressants (*n* = 7) after five (and 10) months. This observation suggests that immunosuppression-induced endothelial changes are possibly reflected by sVCAM-1 serum concentrations. However, also in this situation, the low number of cases does not permit postulating a class-specific immunosuppressant effect. Similar observations have been described for plasma sVCAM-1 concentrations after treatment with imatinib [46], prostanoids [47], and nifedipine [48]. The numerical decline of ANA titers in our study during immunosuppression may additionally indicate a systemic effect on plasma cell activity. Our patients did not have severe organ involvement, such as extensive pulmonary arterial hypertension, pulmonary fibrosis, or DUs. The decline in sVCAM-1 suggests that activated endothelium may perhaps specifically respond to a reduction of immune and plasma cell activity. A large prospective study on patients with highly active diseases, such as PAH, DU, pulmonary fibrosis, or renal involvement, would be necessary to confirm our results and perhaps validate sVCAM-1 as a disease marker.

This work as also been presented in the form of a poster at the annual meeting 2015 of the European League Against Rheumatism. [49].

## 5. Conclusion

Our study shows a strong signal in a defined set of ANA^+^ patients. As sVCAM-1 can be measured by commercially available ELISA kits, it may be useful for the clinician as an alternative, reliable, and objective serum biomarker. Further studies are underway to prospectively validate and perhaps establish this marker in rheumatology.

## Figures and Tables

**Table 1:** Serological, clinical, and other diagnostic findings in patients with first-time determined positive serum ANA and musculoskeletal complaints.

Diagnoses in patients with positive ANA	Age ± SD (years)	ESR ± SD (mm)	SCL-70/ACA (*n*)	CRP ± SD (mg/l)	ANA (titer) median	ECHO- PAP ≥ 30 mmHg (*n*, %)	FEV1% VC	VC	TLCO/VA ≤ 70% (*n*)	RP (*n*)	Mean HAQ score ± SD	Mean VAS ± SD (mm)	Physician Global Assessment Scale 0–10	Patient Global Assessment scale 0–10
RA *n* = 32	51.9 ± 13.9	20.1 ± 24.1	0 (0%)	6.9 ± 12.8	1 : 320	2 (6.25%)	109.2 ± 9.9	98.7 ± 15.9	8 (25%)	9 (28%)	1.0 ± 0.8	53.1 ± 28.6	5.5 ± 2.1	6.0 ± 2.5
UCTD *n* = 30	54 ± 14	15 ± 13	0 (0%)	4.6 ± 3.8	1 : 320	3 (10%)	111.0 ± 15.6	88.5 ± 15.4	9 (30%)	2 (7%)	0.3 ± 0.3	33.5 ± 26.4	3.0 ± 2.0	3.5 ± 2.3
SSc *n* = 11	56 ± 13.1	18.5 ± 19.9	11 (100%)	5,8 ± 7.8	1 : 10240	3 (27%)	109.6 ± 9.7	94.0 ± 13.7	2 (18%)	9 (82%)	0.5 ± 0.6	42 ± 31.2	4.6 ± 2.6	5.2 ± 3.17
SjS *n* = 7	45 ± 13	19 ± 16	0 (0%)	8,2 ± 14.1	1 : 320	0 (0%)	105.8 ± 6.8	86.7 ± 17.0	1 (14%)	1 (14%)	0.7 ± 0.7	37.8 ± 30.8	5.0 ± 2.8	5.1 ± 2.9

Findings are shown as mean with exception to ANA titers that are indicated as median. ECHO, FEV1% VC, TLCO VA, and RP are indicated as %. RA: rheumatoid arthritis; UCTD: undifferentiated connective tissue diseases; SSc: systemic sclerosis; SjS: Sjörgen's syndrome; ESR: erythrocyte sedimentation rate; ACA: anti-SCL-70 or anti-centromere antibodies; CRP: C-reactive protein.

**Table 2:** Clinical findings in patients with SSc that received immunosuppression (*n* = 7), measured at baseline and after 3, 5, and 10 months (mean ± SD or median) of treatment.

	ESR (mm)	CRP mg/l	HAQ score	VAS (mm)	ECHO-PAP (mmHg)	TLCO/VA	ANA titer
Baseline	18.55 ± 17.8	5.8 ± 7.8	0.5 ± 0.6	42.4 ± 31.2	20.1 ± 8.9	87.4 ± 20.7	1 : 10240
3 months	23.0 ± 32.2	5.1 ± 10.4	0.9 ± 0.3	59.6 ± 20.9	n. d.	n. d.	1 : 5120
5 months	11.4 ± 13.1	3.1 ± 7.1	1.2 ± 0.9	58.7 ± 12.3	18.3 ± 6.2	92.6 ± 17.8	1 : 1280
10 months	9.4 ± 10.7	3.1 ± 6.4	1.1 ± 0.5	53.6 ± 13.5	18.5 ± 6.4	88.4 ± 17.0	1 : 1280

Findings are shown as means with exception to the ANA titer that is indicated as median. ANA numerically declined during immunosuppression. Four patients (not shown) did not require medication, due to low clinical disease activity. ESR: erythrocyte sedimentation rate; CRP: C-reactive protein; ANA: antinuclear antibodies; ECHO-PAP: pulmonary-arterial pressure determined by echocardiography; TLCO/VA: pulmonary diffusion capacity; RP: Raynaud phenomenon; HAQ: Health Assessment Questionnaire: VAS: Visual Analogue Pain Scale.

## References

[1] Giannouli E., Chatzidimitriou D., Gerou S., Gavriilaki E., Settas L., Diza E. (2013). Frequency and specificity of antibodies against nuclear and cytoplasmic antigens in healthy individuals by classic and new methods. *Clinical Rheumatology*.

[2] Peter J. V., Griffith M. F., Prakash J. A. J., Chrispal A., Pichamuthu K., Varghese G. M. (2014). Anti-nuclear antibody expression in severe scrub typhus infection: preliminary observations. *Journal of Global Infectious Diseases*.

[3] O’Sullivan M., McLean-Tooke A., Loh R. (2013). Antinuclear antibody test. *Australian Family Physician*.

[4] Koenig M., Dieudé M., Senécal J.-L. (2008). Predictive value of antinuclear autoantibodies: the lessons of the systemic sclerosis autoantibodies. *Autoimmunity Reviews*.

[5] Ho K. T., Reveille J. D. (2003). The clinical relevance of autoantibodies in scleroderma. *Arthritis Research & Therapy*.

[6] Sulli A., Ruaro B., Smith V. (2013). Progression of nailfold microvascular damage and antinuclear antibody pattern in systemic sclerosis. *The Journal of Rheumatology*.

[7] Koch A. E., Distler O. (2007). Vasculopathy and disordered angiogenesis in selected rheumatic diseases: Rheumatoid arthritis and systemic sclerosis. *Arthritis Research & Therapy*.

[8] McMurray R. W. (1996). Adhesion molecules in autoimmune disease. *Seminars in Arthritis and Rheumatism*.

[9] Seron D., Cameron J. S., Haskard D. O. (1991). Expression of VCAM-1 in the normal and diseased kidney. *Nephrology Dialysis Transplantation *.

[10] Clark E. A., Grabstein K. H., Shu G. L. (1992). Cultured human follicular dendritic cells: Growth characteristics and interactions with B lymphocytes. *The Journal of Immunology*.

[11] Wilkinson L. S., Edwards J. C. W., Poston R. N., Haskard D. O. (1993). Expression of vascular cell adhesion molecule-1 in normal and inflamed synovium. *Laboratory Investigation*.

[12] Needleman B. W. (1990). Increased expression of intercellular adhesion molecule 1 on the fibroblasts of scleroderma patients. *Arthritis & Rheumatism*.

[13] Bevilacqua M. P., Stengelin S., Gimbrone M. A., Seed B. (1989). Endothelial leukocyte adhesion molecule 1: an inducible receptor for neutrophils related to complement regulatory proteins and lectins. *Science*.

[14] Sugama Y. (1992). Thrombin-induced expression of endothelial P-selectin and intercellular adhesion molecule-1: a mechanism for stabilizing neutrophil adhesion. *The Journal of Cell Biology*.

[15] Distler O., Del Rosso A., Giacomelli R. (2002). Angiogenic and angiostatic factors in systemic sclerosis: increased levels of vascular endothelial growth factor are a feature of the earliest disease stages and are associated with the absence of fingertip ulcers. *Arthritis Research & Therapy*.

[16] Coll-Vinent B., Vilardell C., Font C. (1999). Circulating soluble adhesion molecules in patients with giant cell arteritis. Correlation between soluble intercellular adhesion molecule-1 (sICAM-1) concentrations and disease activity. *Annals of the Rheumatic Diseases*.

[17] Yamane K., Ihn H., Kubo M. (2000). Increased serum levels of soluble vascular cell adhesion molecule 1 and E-selectin in patients with localized scleroderma. *Journal of the American Academy of Dermatology*.

[18] Kaplanski G., Cacoub P., Farnarier C. (2000). Increased soluble vascular cell adhesion molecule 1 concentrations in patients with primary or systemic lupus erythematosus-related antiphospholipid syndrome: correlations with the severity of thrombosis. *Arthritis & Rheumatology*.

[19] Bečvář R., Štork J., Pešáková V. (2005). Clinical correlations of potential activity markers in systemic sclerosis. *Annals of the New York Academy of Sciences*.

[20] Navarro-Hernández R. E., Oregon-Romero E., Mercado M. V.-D., Rangel-Villalobos H., Palafox-Sánchez C. A., Muoz-Valle J. F. (2009). Expression of ICAM1 and VCAM1 serum levels in rheumatoid arthritis clinical activity. Association with genetic polymorphisms. *Disease Markers*.

[21] Blann A. D., Herrick A., Jayson M. I. V. (1995). Altered levels of soluble adhesion molecules in rheumatoid arthritis, vasculitis and systemic sclerosis. *Rheumatology*.

[22] Ihn H., Sato S., Fujimoto M., Takehara K., Tamaki K. (1998). Increased serum levels of soluble vascular cell adhesion molecule-1 and E-selectin in patients with systemic sclerosis. *British Journal of Rheumatology*.

[23] Gruschwitz M. S., Hornstein O. P., Driesch P. V. D. (1995). Correlation of soluble adhesion molecules in the peripheral blood of scleroderma patients with their in situ expression and with disease activity. *Arthritis & Rheumatism*.

[24] Kahaleh B. (2008). Vascular disease in scleroderma: mechanisms of vascular injury. *Rheumatic Disease Clinics of North America*.

[25] Muangchan C., Harding S., Khimdas S., Bonner A., Baron M., Pope J. (2012). Association of C-reactive protein with high disease activity in systemic sclerosis: results from the Canadian Scleroderma Research Group. *Arthritis Care & Research*.

[26] Valentini G., D'Angelo S., Della Rossa A., Bencivelli W., Bombardieri S. (2003). European Scleroderma Study Group to define disease activity criteria for systemic sclerosis. IV. Assessment of skin thickening by modified Rodnan skin score. *Annals of the Rheumatic Diseases*.

[27] Aletaha D., Neogi T., Silman A. J. (2010). Rheumatoid arthritis classification criteria: an American College of Rheumatology/European League Against Rheumatism collaborative initiative. *Arthritis & Rheumatism*.

[28] Van den Hoogen F., Khanna D., Fransen J. (2013). classification criteria for systemic sclerosis: an American College of Rheumatology/European league against Rheumatism collaborative initiative. *Arthritis & Rheumatology*.

[29] Hochberg M. C. (1997). Updating the American College of Rheumatology revised criteria for the classification of systemic lupus erythematosus. *Arthritis & Rheumatology*.

[30] Vitali C., Bombardieri S., Jonsson R. (2002). Classification criteria for Sjögren’s syndrome: a revised version of the European criteria proposed by the American-European consensus group. *Annals of the Rheumatic Diseases*.

[31] Mosca M., Neri R., Bombardieri S. (1999). Undifferentiated connective tissue diseases (UCTD): A review of the literature and a proposal for preliminary classification criteria. *Clinical and Experimental Rheumatology*.

[32] Volkmann E. R., Taylor M., Ben-Artzi A. (2012). Using the antinuclear antibody test to diagnose rheumatic diseases: when does a positive test warrant further investigation?. *Southern Medical Journal*.

[33] Walravens M. (1987). Systemic diseases and the detection of antinuclear and anticytoplasmic antibodies. An hystorical review. *Clinical Rheumatology*.

[34] Kubo M., Ihn H., Yamane K. (2000). Increased serum levels of soluble vascular cell adhesion molecule-1 and soluble E-selectin in patients with polymyositis/dermatomyositis. *British Journal of Dermatology*.

[35] Spronk P. E., Bootsma H., Huitema M. G., Limburg P. C., Kallenberg C. G. M. (1994). Levels of soluble VCAM-1, soluble ICAM-1, and soluble E-selectin during disease exacerbations in patients with systemic lupus erythematosus (SLE); a long term prospective study. *Clinical & Experimental Immunology*.

[36] Cuida M., Halse A.-K., Johannessen A. C., Tynning T., Jonsson R. (1997). Indicators of salivary gland inflammation in primary Sjögren’s syndrome. *European Journal of Oral Sciences*.

[37] Herrick A. L., Illingworth K., Blann A., Hay C. R. M., Hollis S., Jayson M. I. V. (1996). Von Willebrand factor, thrombomodulin, thromboxane, *β*-thromboglobulin and markers of fibrinolysis in primary Raynaud’s phenomenon and systemic sclerosis. *Annals of the Rheumatic Diseases*.

[38] Cerinic M. M., Valentini G., Sorano G. G. (2003). Blood coagulation, fibrinolysis, and markers of endothelial dysfunction in systemic sclerosis. *Seminars in Arthritis and Rheumatism*.

[39] Kuryliszyn-Moskal A., Klimiuk P. A., Sierakowski S. (2005). Soluble adhesion molecules (sVCAM-1, sE-selectin), vascular endothelial growth factor (VEGF) and endothelin-1 in patients with systemic sclerosis: relationship to organ systemic involvement. *Clinical Rheumatology*.

[40] Davies C. A., Jeziorska M., Freemont A. J., Herrick A. L. (2006). The differential expression of VEGF, VEGFR-2, and GLUT-1 proteins in disease subtypes of systemic sclerosis. *Human Pathology*.

[41] Hummers L. K. (2006). Microvascular damage in systemic sclerosis: detection and monitoring with biomarkers. *Current Rheumatology Reports*.

[42] Castro S. V., Jimenez S. A. (2010). Biomarkers in systemic sclerosis. *Biomarkers in Medicine*.

[43] Denton C. P., Bickerstaff M. C. M., Shiwen X. (1995). Serial circulating adhesion molecule levels reflect disease severity in systemic sclerosis. *Rheumatology*.

[44] Kiener H., Graninger W., Machold K., Aringer M., Graninger W. B. (1994). Increased levels of circulating intercellular adhesion molecule-1 in patients with systemic sclerosis. *Clinical and Experimental Rheumatology*.

[45] Machold K. P., Kiener H. P., Graninger W., Graninger W. B. (1993). Soluble intercellular adhesion molecule-1 (sICAM-1) in patients with rheumatoid arthritis and systemic lupus erythematosus. *Clinical Immunology and Immunopathology*.

[46] Pope J., Walker K. M., de Leon F., Vanderhoek L., Seney S., Summers K. L. (2014). Correlations between changes in cytokines and clinical outcomes for early phase (proof of concept) trials in active diffuse systemic sclerosis using data from an imatinib study. *Rheumatology*.

[47] Rehberger P., Beckheinrich-Mrowka P., Haustein U.-F., Sticherling M. (2009). Prostacyclin analogue iloprost influences endothelial cell-associated soluble adhesion molecules and growth factors in patients with systemic sclerosis: a time course study of serum concentrations. *Acta Dermato-Venereologica*.

[48] Allanore Y., Borderie D., Lemaréchal H., Ekindjian O. G., Kahan A. (2004). Nifedipine decreases sVCAM-1 concentrations and oxidative stress in systemic sclerosis but does not affect the concentrations of vascular endothelial growth factor or its soluble receptor 1. *Arthritis Research & Therapy*.

[49] Oleszowsky M., Seidel M. (2015). Soluble vascular cell adhesion molecule-1 is overexpressed as a disease marker in patients with first-time diagnosed antinuclear antibodies and significantly decreases after immunosuppression in patients with systemic sclerosis, annals of the rheumatic Diseases. *Annals of The Rheumatic Diseases*.

